# Determination of Fetal State from Cardiotocogram Using LS-SVM with Particle Swarm Optimization and Binary Decision Tree

**DOI:** 10.1155/2013/487179

**Published:** 2013-10-29

**Authors:** Ersen Yılmaz, Çağlar Kılıkçıer

**Affiliations:** Electrical-Electronic Engineering Department, Uludag University, 16059 Gorukle, Bursa, Turkey

## Abstract

We use least squares support vector machine (LS-SVM) utilizing a binary decision tree for classification of cardiotocogram to determine the fetal state. The parameters of LS-SVM are optimized by particle swarm optimization. The robustness of the method is examined by running 10-fold cross-validation. The performance of the method is evaluated in terms of overall classification accuracy. Additionally, receiver operation characteristic analysis and cobweb representation are presented in order to analyze and visualize the performance of the method. Experimental results demonstrate that the proposed method achieves a remarkable classification accuracy rate of 91.62%.

## 1. Introduction

There is a growing tendency to use clinical decision support systems in medical diagnosis. These systems help to optimize medical decisions, improve medical treatments, and reduce financial costs [1, 2]. A large number of the medical diagnosis procedures can be converted into intelligent data classification tasks. These classification tasks can be categorized as two-class task and multiclass task. The first type separates the data between only two classes while the second type involves the classification of the data with more than two classes [3].

Cardiotocography was introduced into obstetrics practice in the early 1970s, and since then it has been used as a worldwide method for antepartum (before delivery) and intrapartum (during delivery) fetal monitoring. Cardiotocogram (CTG) is a recording of two distinct signals, fetal heart rate (FHR), and uterine activity (UA) [4]. It is used for determining the fetal state during both pregnancy and delivery. The aim of the CTG monitoring is to determine babies who may be short of oxygen (hypoxic); thus further assessments of fetal condition may be performed or the baby might be delivered by caesarean section or natural birth [5]. The visual evaluation of the CTG not only requires time but also depends on the knowledge and clinical experience of obstetricians. 

A clinical decision support system eliminates the inconsistency of visual evaluation. There have been proposed several classification tools for developing such system [4, 6–10]. 

One of these tools is support vector machine (SVM) and it is used in [4, 8, 10]. In [4, 8], SVM is used for FHR signal classification with two classes, normal or at risk. The risk of metabolic acidosis for newborn based on FHR signal is predicted in [4] while the classification of antepartum FHR signal is made in [8]. In [10], a medical decision support system based on SVM and genetic algorithm (GA) is presented for the evaluation of fetal well-being from the CTG recordings as normal or pathologic.

In [6], an approach based on hidden Markov models (HMM) is presented for automatic classification of FHR signal belonging to hypoxic and normal newborns. In [7], an ANBLIR (Artificial Neural Network Based on Logical Interpretation of fuzzy if-then Rules) system is used to evaluate the risk of low-fetal birth weight as normal or abnormal using CTG signals recorded during the pregnancy.

In [9], an adaptive neurofuzzy inference system (ANFIS) is proposed for the prediction of fetal state from the CTG recordings as normal or pathologic.

Support vector machines (SVM) is developed for two-class task, but classification problems generally require multi-class task. There are several methods proposed in the literature based on binary decision tree (BDT) to extend the binary SVMs to multi-class problems, for example, [11, 12].

LS-SVM is a modified version of SVM in a least square sense [13]. The higher computational load of SVM is overcome by LS-SVM because LS-SVM solves the problem using a set of linear equations while SVM solves as a quadratic programming problem. 

The choice of appropriate kernel function and the model parameters (including kernel parameters) is crucial for SVM-based methods, and this influences directly the classification performance. The most common kernel functions used in the literature are polynomial, Gaussian radial basis, exponential radial basis, and sigmoid. 

Performance evaluation of classifiers is a fundamental step for determining the best classifier or the best set of parameters for a classifier [14]. In general, the overall classification accuracy is a natural way to measure the performance of the classifiers. The classifier predicts the class for each data point in the data set; if the prediction is correct it is counted as a success and if it is wrong it is counted as an error. The overall classification accuracy is computed as the ratio of the number of successes over the number of the whole data points to be classified. 

For many classification problems, especially in the medical diagnosis, the overall classification accuracy is not adequate alone because in general not all errors have the same consequences. Wrong diagnoses can cause different cost and dangers depending on which kind of mistakes have been done [15]. Therefore, for such situations, in addition to overall classification accuracy receiver operation characteristic (ROC) analysis is usually performed [16]. 

In this paper, we use LS-SVM utilizing a BDT for classification of the CTG data to determine the fetal state as normal, suspect, or pathologic. Gaussian radial basis function is chosen as the kernel of LS-SVM, and the model parameters, which are the penalty factor and the width of Gaussian kernel, are optimized by using particle swarm optimization (PSO). The robustness of the proposed method LS-SVM-PSO-BDT is examined with 10-fold cross-validation (10-fold CV) on the CTG data set taken from UCI machine learning repository. The performance of the method is evaluated in terms of overall classification accuracy. Additionally, ROC analysis and cobweb representation are presented in order to analyze and visualize the performance of the method.

## 2. Support Vector Machine (SVM)

SVM is a powerful supervised learning algorithm based on statistical learning theory that has been widely used for solving a wide range of data classification problems since it was first introduced by Boser et al. [17]. SVM builds a hyperplane separating the data points into two different classes with a maximum margin. 

A given training set of *N* data points (*x*_*i*_, *y*_*i*_), *x*_*i*_ ∈ *R* ^*p*^, and *y*_*i*_ ∈ ±1, where *x*_*i*_ is a data point and *y*_*i*_ is the corresponding class label; SVM requires the minimization of the following primal optimization problem:

(1) min⁡w,b,ξJ(w,ξ)=12||w||2+C∑i=1Nξisubject toyi(wTφ(xi)+b)≥1−ξi, i=1,…,N,

where *w* is the normal vector to hyperplane, *b* is the bias or offset scalar, *ξ*_*i*_ are the slack parameters which are used to allow soft margins, *C* is the penalty parameter which controls the trade-off between minimizing the error and maximizing the margin, and *φ*(*x*_*i*_) is a nonlinear mapping from the input space to the higher dimensional feature space [4, 8, 13, 17, 18].

The corresponding dual problem of (1) is given by

(2) max⁡αJ(α)=∑i=1Nαi−12∑i=1N∑j=1NαiαjyiyjK(xi,xj) subject to∑i=1Nαiyi=0, 0≤αi≤C,  ∀i,

where *α*_*i*_ are Lagrange multipliers, the term *K*(*x*_*i*_, *x*_*j*_) is a kernel function representing the inner product of two vectors in the feature space, that is, *φ* ^*T*^(*x*_*i*_)*φ*(*x*_*j*_). Kernel function must satisfy the well-known Mercer's condition. The data points for which *α*_*i*_ > 0 are called support vectors, which construct the following decision function [4, 8, 13, 17, 18]:

(3) $$\begin{array}{c} f\left(x\right)=\mathrm{sign}\left(\sum_{i=1}^{N}y_{i}\alpha_{i}K\left(x,x_{i}\right)+b\right), \end{array}$$

where *b* = −(1/2)∑_*i*=1_^*N*^ *y*_*i*_ *α*_*i*_(*K*(*x*_+_, *x*_*i*_) + *K*(*x*_−_, *x*_*i*_)), *x*_+_ and *x*_−_ are two arbitrary supporting vectors from different classes *y*_*i*_ ∈ ±1 [17]. 

## 3. Least Squares SVM (LS-SVM)

LS-SVM is originally proposed by Suykens and Vandewalle as a modification to SVM regression formulation [13]. The idea behind the modification is to transform the problem from a quadratic programming problem to solving a set of linear equations.

The optimization problem has been modified as follows:

(4) min⁡w,b,eJLS(w,e)=12||w||2+12γ∑i=1Nei2subject to  yi(wTφ(xi)+b)=1−ei, i=1,…,N,

where *γ* and *e*_*i*_ are similar to the penalty parameter *C* and the slack variable *ξ*_*i*_ of SVM, respectively. In (4), it can be easily seen that the following two modifications are made; the first one is that the inequality constraints are replaced by the equality constraints, and the second one is that the squared loss function is taken for *e*_*i*_. These modifications significantly simplify the problem [19].

To solve the optimization problem in (4), Lagrangian function is defined as given below:

(5) LLS(w,b,e;α)=JLS(w,e)  −∑i=1Nαi{yi[wTφ(xi)+b]−1+ei},

where *α*_*i*_ are Lagrange multipliers, which can be positive or negative due to the equality constraints. According to optimality conditions, we can get

(6) ∂LLs∂w=0, w=∑i=1Nαiyiφ(xi), ∂LLs∂b=0, ∑i=1Nαiyi=0,∂LLs∂ei=0, αi=γei, i=1,…,N,∂LLs∂αi=0, yi[wTφ(xi)+b]−1+ei=0, i=1,…,N.

Defining *Z* = [*φ* ^*T*^(*x*_1_)*y*_1_; …; *φ* ^*T*^(*x*_*N*_)*y*_*N*_], *Y* = [*y*_1_; …; *y*_*N*_], *I* = [1; …; 1], *e* = [*e*_1_; …; *e*_*N*_], *α* = [*α*_*i*_; …; *α*_*N*_] and after elimination of *w* and *e*, a linear Karush-Kuhn-Tucker system is obtained as in (7) [13]:

(7) $$\begin{array}{c} \left[\begin{array}{c} \underline{0\;|\;\begin{array}{c} -Y^{T} \end{array}}\\ Y\;|\;\begin{array}{c} \Omega +\gamma^{-1}I \end{array} \end{array}\right]\left[\begin{array}{c} \underline{b}\\ \alpha \end{array}\right]=\left[\begin{array}{c} \underline{0}\\ I \end{array}\right], \end{array}$$

where Ω = *ZZ* ^*T*^ and the Mercer's condition can be applied to the matrix Ω:

(8) $$\begin{array}{c} \Omega_{i,j}=y_{i}y_{j}\varphi {\left(x_{i}\right)}^{T}\varphi \left(x_{j}\right)=y_{i}y_{j}K\left(x_{i},x_{j}\right),\;i,j=1,\ldots ,N. \end{array}$$

LS-SVM classifier takes the form as in (9) which is similar to SVM case as in (3) and found by solving the linear set of equations in (7):

(9) $$\begin{array}{c} f\left(x\right)=\mathrm{sign}\left(\sum_{i=1}^{N}y_{i}\alpha_{i}K\left(x,x_{i}\right)+b\right). \end{array}$$

## 4. Particle Swarm Optimization (PSO)

PSO is a swarm intelligence based optimization method proposed by Kennedy and Eberhart inspired by social behavior of bird flocking and fish schooling [20]. In PSO, the procedure begins with an initialization step in which a population (swarm) of possible solutions (particles) is chosen in the search space and then searches for optimum solution by updating particles over generations. 

The particles are updated by iteratively by using the following equations:

(10) $$\begin{array}{c} V_{i}^{k+1}=\omega V_{i}^{k}+c_{1}r_{1}^{k}\left(P_{i}^{k}-\lambda_{i}^{k}\right)+c_{2}r_{2}^{k}\left(G^{k}-\lambda_{i}^{k}\right)\\ \lambda_{i}^{k+1}=\lambda_{i}^{k}+V_{i}^{k+1}, \end{array}$$

where *λ*_*i*_ = [*λ*_*i*1_,…, *λ*_*iM*_] and *V*_*i*_ = [*V*_*i*1_,…, *V*_*iM*_] are the current position and the velocity of the *i*th particle in *M* dimensional space and *G* = [*G*_1_,…, *G*_*M*_] and *P*_*i*_ = [*P*_*i*1_,…, *P*_*iM*_] are the best position of the swarm and the best position of the *i*th particle, respectively.

The value of inertia weight *ω* is a trade-off between global search and local search. A bigger value of inertia weight allows the particles to search new areas in the search space (global search) while a smaller value let the particles move in the current search area for fine tuning (local search). The cognitive and the social learning factors *c*_1_ and *c*_2_ are positive constants, and *r*_1_ and *r*_2_ are random numbers in the range [0,1] [20, 21].

## 5. Binary Decision Tree (BDT)

**Figure 1:**
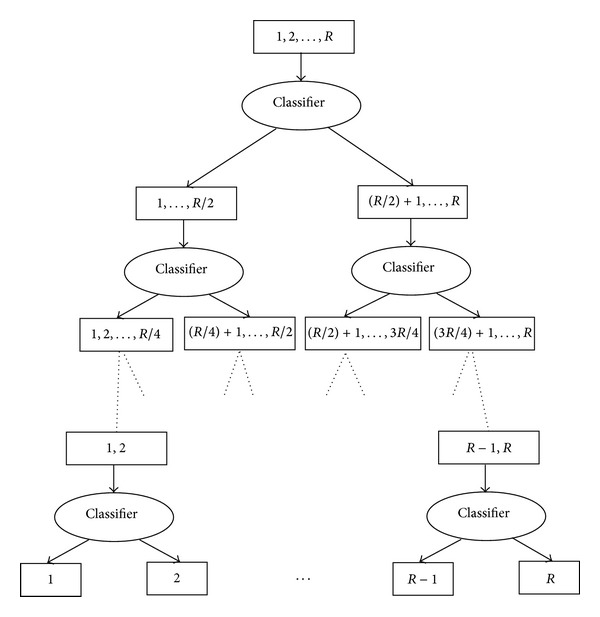
BDT architecture for classification of data set with *R* classes.

BDT architecture for classification of data sets with *R* classes requires *R* − 1 classifiers. The architecture for classification of a data set with *R* classes is shown in Figure 1. There is a classifier at each node in the tree to make a binary decision.

## 6. Cross-Validation (CV)

CV is a most commonly used statistical method for evaluating and comparing the learning algorithms by separating the data set into two sets as training and testing. In CV, the training and testing sets must cross-over in successive rounds, and thus each data point has a chance of being validated against [22]. 

General form of CV is *k*-fold CV in which the data set is divided into *k* groups of (almost) equal size, and *k* iterations are made. In each iteration step, one of the *k*  groups is used for testing and the remaining *k* − 1 groups are used for training. 

## 7. ROC Analysis

ROC analysis has been used a standard tool for the design, optimization, and evaluation of two-class classifiers [23]. In ROC analysis with two classes, the notation, which is given in Table 1, is used for the confusion matrix [24].

ROC analysis investigates and employs the relationship between sensitivity and specificity of two-class classifiers while decision threshold varies [25]. Sensitivity is the true positive rate while specificity is the true negative rate, and they are defined as TP/(TP+FN) and TN/(TN+FP), respectively [24]. 

ROC curve represents the performance of a classifier in a two-dimensional graph, and conventionally the true positive rate is plotted against the false positive rate [25]. Detailed information about ROC analysis can be found in [23–28]. 

The extension of ROC analysis for more than two classes has been studied extensively in the literature [15, 23, 27, 29, 30]. For *R* classes, the confusion matrix is *R* × *R* matrix such that its diagonal entries contain the *R* correct classifications while its off-diagonal entries contain *R* ^2^ − *R* possible errors. Therefore, generating ROC curves for visualizing the performance of a classifier becomes difficult as the number of classes increase, for example, a six-dimensional space is required for three classes. Recently, cobweb representation is used to visualize the performance of the classifiers in the form of multiclass version of ROC analysis [30]. 

## 8. Cobweb Representation

**Figure 2:**
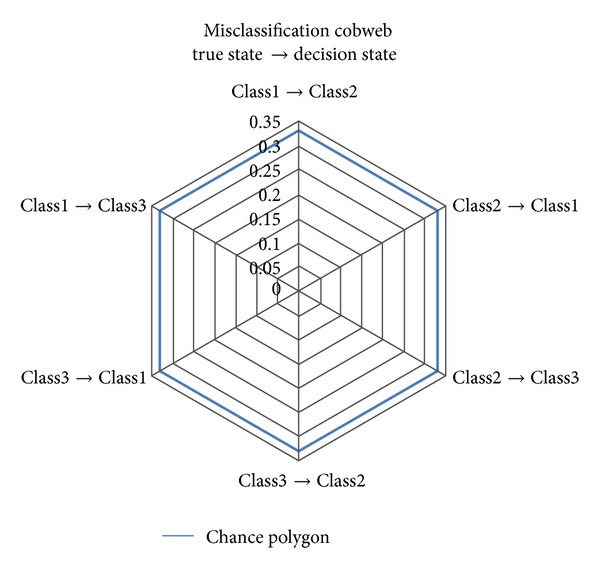
Misclassification cobweb for a chance classification with three classes.

The cobweb representation is generated by using the misclassification ratios of the confusion ratio matrix, which is column-normalized version of the confusion matrix. Let us consider a chance classification with *R* classes. The confusion ratio matrix has *R* ^2^ − *R* misclassification rates which are equal to 1/*R*. The misclassification rates of 1/*R* show that when confronted with a data point from one of the classes the classifier classifies it as having the same chances of being from any of *R* classes. A polygon with *R* ^2^ − *R* equal sides can be formed to map the misclassification rates of the confusion ratio matrix. This polygon (chance polygon) is used to compare the performance of any classifier with the chance classifier in terms of misclassification rates. Any polygon within the chance performance polygon shows a better performance than chance performance. For a chance classification with three classes, the misclassification rates are (0.33, 0.33, 0.33, 0.33, 0.33, 0.33), and the chance polygon becomes a hexagon given as in Figure 2 [30, 31].

## 9. CTG Data Set 

The CTG data set used in this study is taken from UCI Machine Learning Repository [http://archive.ics.uci.edu/ml/datasets/Cardiotocography], (last accessed: June, 2013) and the details can be found in [32]. This data set has 2126 data points from three classes representing the fetal state as normal, suspect, or pathologic. All data points have 21 features, and these features are listed in Table 2. 

## 10. Proposed LS-SVM-PSO-BDT Method

**Figure 3:**
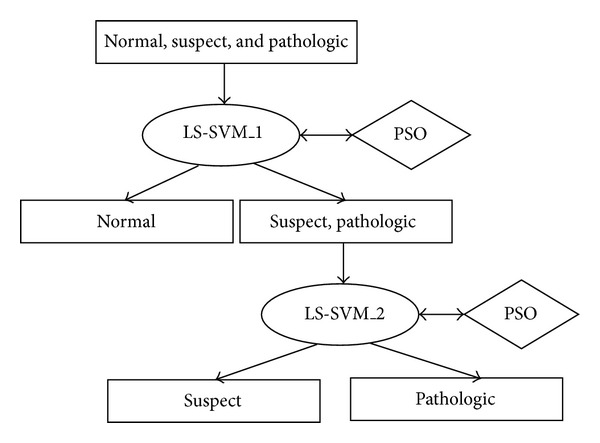
The proposed method's architecture.

The proposed LS-SVM-PSO-BDT method for fetal state determination is described in this section. Its architecture is given in Figure 3.

There are two nodes in BDT due to that the CTG data has three classes. A Gaussian radial basis function, which is illustrated in (11), is chosen as the kernel function of LS-SVMs:

(11) $$\begin{array}{c} K\left(x,x_{i}\right)=\exp\left(-\frac{1}{2\sigma^{2}}{\left(x-x_{i}\right)}^{2}\right), \end{array}$$

where *σ* ^2^ is the width of the kernel.

LS-SVM parameters, the penalty factor *γ*, and the kernel width *σ* ^2^ are optimized by using PSO.

Training procedure of the method is summarized as the following sequential steps.

**Step 1**

Training data points are put into the root node and divided into two groups as PS (pathologic and suspect) and Nr (normal).

**Step 2**

LS-SVM_ 1 is trained on the data points in the root node to classify the data points as PS or Nr. Meanwhile LS-SVM_ 1 parameters are optimized by using PSO.

**Step 3**

LS-SVM_ 2 is trained on the data points in the subnode PS to classify the data points as P (pathologic) or S (suspect). Meanwhile, LS-SVM_ 2 parameters are optimized by using PSO.

In the first step, the reason why we combine pathologic and suspect data points in one group instead of combining normal and suspect data points is to minimize the risk of making decisions that cause abnormalities in babies.

## 11. Experimental Results and Discussions

The proposed method LS-SVM-PSO-BDT is used for the classification of the CTG data set which is taken from the UCI Machine Learning Repository. 

In order to validate the robustness of the method a 10-fold CV procedure is performed. The entire data set is randomly divided into ten subsets of approximately equal size while keeping the proportion of data points from different classes in each subset roughly the same as that in the whole data set. In each fold, one subset is left out for testing, and the union of the remaining nine sets is used for training. Thus, after ten folds, each subset is used once for testing purpose. The final result is average result of these ten folds.

In the experiment, the parameters for LS-SVM-PSO-BDT are set as follows. Twenty-five particles are used in PSOs. The initial values of 25 particles for the penalty factor *γ* and the kernel width *σ* ^2^ are chosen on the intervals *γ*, *σ* ^2^ ∈ [2^−4^, 212].

The inertia weight, cognitive, and social learning factors of PSOs are chosen as *ω* = 0.75, *c*_1_ = 2, and *c*_2_ = 2. The codes for the proposed method have been developed in MATLAB [33], without using any toolbox. The classification accuracies for ten folds are reported in Table 3.

The overall classification accuracy of LS-SVM-PSO-BDT, which is average accuracy of ten folds, is obtained as 91.62%. 

There have been similar works focusing on the classification of the CTG data in the literature [4, 6–10]. It is not possible to make a direct comparison of the methods in these works with the proposed method because they are all used for two-class task and additionally the properties of the CTG data sets used in [4, 6–8] are different. But, based on the overall classification accuracy, a comparison of the proposed method with the methods used in above mentioned works is provided in Table 4.

Although the number of classes and the number of data points in the CTG data set used in our work are larger than those in above mentioned works, LS-SVM-PSO-BDT achieves a remarkable classification accuracy rate of 91.62%.

In addition to overall classification accuracy ROC methodology is used to analyze the performance of the method in more detail. Therefore, a confusion matrix is created to analyze the classification results, which is given in Table 5. This table shows the number of correctly and incorrectly classified data points from the CTG data.

In order to visualize the performance of the proposed method a cobweb representation is presented. Cobweb representation is generated by using the misclassification ratios from the confusion ratio matrix, which is column-normalized version of the confusion matrix. The confusion ratio matrix of the proposed method is given in Table 6.

Diagonal entries of the confusion ratio matrix show the correct classification ratios while its off-diagonal entries show the misclassification ratios. From Table 6, 96.90% of normal data points, 70.50% of suspect data points, and 76.70% of pathologic data points are correctly classified as normal, suspect, and pathologic, respectively.

**Figure 4:**
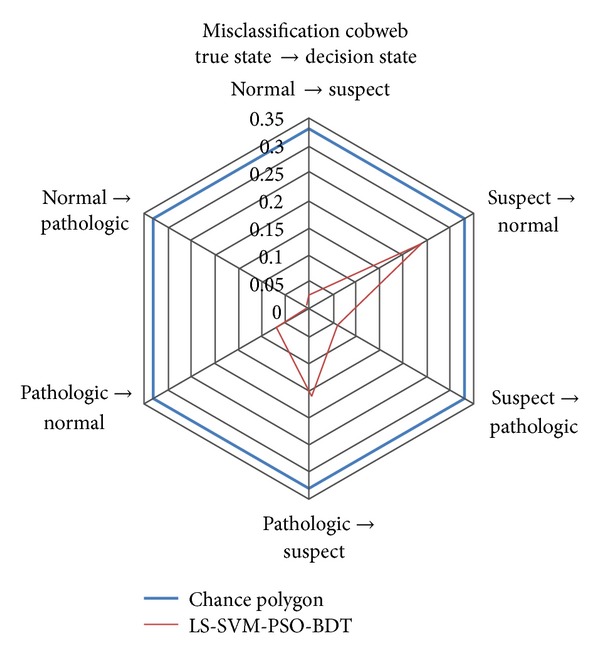
Misclassification cobweb for LS-SVM-PSO-BDT.

Cobweb representation of the proposed method is given in Figure 4. It can be seen from Figure 4 that the misclassification ratios of LS-SVM-PSO-BDT are smaller than those of the chance classifier.

## 12. Conclusions

In this work, we use LS-SVM utilizing a BDT for classification of the CTG data to determine the fetal state as normal, suspect, or pathologic. Gaussian radial basis function is chosen as the kernel of LS-SVM, and the model parameters, which are the penalty factor and the width of Gaussian kernel, are optimized by using PSO. The robustness of LS-SVM-PSO-BDT is examined by running 10-fold CV. The performance of the proposed method is evaluated in terms of overall classification accuracy. According to empirical results, the proposed LS-SVM-PSO-BDT method achieves a remarkable overall classification accuracy rate of 91.62%.

Additionally, ROC methodology is used to analyze the performance of the method in more detail. The correct classification and misclassification ratios of the method with the respect to each individual class are presented. 96.90% of normal data points, 70.50% of suspect data points, and 76.70% of pathologic data points are correctly classified as normal, suspect, and pathologic, respectively. In order to visualize the performance of the method, a cobweb representation is presented. This representation indicates that misclassification ratios of the proposed method are smaller than those of the chance classifier. Empirical results show that the proposed method can help the obstetricians to make more accurate decision in determining the fetal state.

## Figures and Tables

**Table 1:** Confusion matrix.

Predicted	Actual	
	Positive	Negative
Positive	TP (true positive)	FP (false positive)
Negative	FN (false negative)	TN (true negative)

**Table 2:** Features used for determining the fetal state.

Features	
LB	FHR baseline (beats per minute)
AC	Number of accelerations per second
FM	Number of fetal movements per second
UC	Number of uterine contractions per second
DL	Number of light decelerations per second
DS	Number of severe decelerations per second
DP	Number of prolonged decelerations per second
ASTV	Percentage of time with abnormal short term variability
MSTV	Mean value of short term variability
ALTV	Percentage of time with abnormal long term variability
MLTV	Mean value of long term variability
Width	Width of FHR histogram
Min	Minimum (low frequency) of FHR histogram
Max	Maximum (high frequency) of FHR histogram
*N*_ max_	Number of histogram peaks
*N*_ zeros_	Number of histogram zeros
Mode	Histogram mode
Mean	Histogram mean
Median	Histogram median
Variance	Histogram variance
Tendency	Histogram tendency

**Table 3:** Classification accuracy for each fold.

Fold-1	Fold-2	Fold-3	Fold-4	Fold-5	Fold-6	Fold-7	Fold-8	Fold-9	Fold-10
89.67%	94.84%	91.08%	94.84%	92.49%	91.55%	88.27%	90.14%	92.96%	90.14%

**Table 4:** Comparison of LS-SVM-PSO-BDT with the existing methods in similar works.

Method	Maximum classification accuracy	Number of classes	Number of data points
LS-SVM-PSO-BDT	91.62%	3	2162

SVM Krupa et al., 2011 [8]	81.50%	2	129

SVM Georgoulas et al., 2006 [4]	81.25%	2	80

Hidden Markov models Georgoulas et al., 2004 [6]	83.00%	2	36

ANBLIR system Czabanski et al., 2010 [7]	97.50%	2	685

ANFIS Ocak and Ertunc, 2012 [9]	97.15%	2	1831

SVM and GA Ocak, 2013 [10]	99.30% (specificity) 100% (sensitivity)	2	1831

**Table 5:** Confusion matrix of LS-SVM-PSO-BDT.

Predicted	Actual		
	Normal	Suspect	Pathologic
Normal	1604	70	12
Suspect	38	208	29
Pathologic	13	17	135

Total	1655	295	176

**Table 6:** Confusion ratio matrix of LS-SVM-PSO-BDT.

Predicted	Actual		
	Normal	Suspect	Pathologic
Normal	0.969	0.237	0.068
Suspect	0.023	0.705	0.165
Pathologic	0.008	0.058	0.767
